# Metabolic and Inflammatory Response in Post-Traumatic Stress Disorder (PTSD): A Systematic Review on Peripheral Neuroimmune Biomarkers

**DOI:** 10.3390/ijerph20042937

**Published:** 2023-02-08

**Authors:** Valerio Dell’Oste, Sara Fantasia, Davide Gravina, Lionella Palego, Laura Betti, Liliana Dell’Osso, Gino Giannaccini, Claudia Carmassi

**Affiliations:** 1 Department of Clinical and Experimental Medicine, University of Pisa, 56126 Pisa, Italy; 2 Department of Biotechnology, Chemistry and Pharmacy, University of Siena, 53100 Siena, Italy; 3 Department of Pharmacy, University of Pisa, 56126 Pisa, Italy

**Keywords:** Post-Traumatic Stress Disorder (PTSD), biomarkers, proinflammatory cytokines, catalase (CAT), superoxide dismutase (SOD), glutathione peroxidase (GPx), tryptophan metabolism, kynurenine, quinolinic acid, melatonin

## Abstract

Several heterogeneous pathophysiology pathways have been hypothesized for being involved in the onset and course of Post-Traumatic Stress Disorder (PTSD). This systematic review aims to summarize the current evidence on the role of inflammation and immunological dysregulations in PTSD, investigating possible peripheral biomarkers linked to the neuroimmune response to stress. A total of 44 studies on the dysregulated inflammatory and metabolic response in subjects with PTSD with respect to controls were included. Eligibility criteria included full-text publications in the English language, human adult samples, studies involving both subjects with a clinical diagnosis of PTSD and a healthy control group. The research was focused on specific blood neuroimmune biomarkers, namely IL-1β, TNF-α, IL-6 and INF-γ, as well as on the potential harmful role of reduced antioxidant activity (involving catalase, superoxide dismutase and glutathione peroxidase). The possible role of the inflammatory-altered tryptophan metabolism was also explored. The results showed conflicting data on the role of pro-inflammatory cytokines in individuals with PTSD, and a lack of study regarding the other mediators investigated. The present research suggests the need for further studies in human samples to clarify the role of inflammation in the pathogenesis of PTSD, to define potential peripheral biomarkers.

## 1. Introduction

According to the fifth version-text revision of the Diagnostical and Statistical Manual for Mental Disorders (DSM-5-TR), Post-Traumatic Stress Disorder (PTSD) is a mental disorder characterized by the onset of typical symptoms following trauma exposure, such as natural disasters, war, rape or sexual abuse [1]. The lifetime prevalence of PTSD varies across studies upon methodological issues, such as the instruments adopted for the assessments, and it is estimated to be around 3.9% worldwide, with double the rates in female compared to male subjects (10–12% vs. 5–6%) [2].

The diagnostic symptoms of PTSD include intrusion symptoms, avoidance, negative alterations in cognition and mood and hyperarousal, lasting more than 1 month, leading to a global functioning impairment and other possible complications as an increased risk for suicide attempts [1,3]. Specifically, intrusion symptoms are characterized by re-experiencing the stressful event, such as dissociative flashbacks and intruding memories that play a key role in the development and persistence of the disorder. However, the clinical manifestations of PTSD are variable, and the majority of people exposed to trauma do not develop the disorder, suggesting that the development of the disease depends not only on the characteristics of the trauma, but also on individuals’ risk factors [4,5,6,7,8].

PTSD is a heterogeneous disorder and multiple pathophysiology pathways are hypothesized to contribute to its onset and endurance. In recent years, the scientific community has explored the possible biological pathways underlying the disorder, highlighting a probable linkage with the hypothalamic–pituitary–adrenal (HPA) axis, autonomic nervous system, monoaminergic transmission system, inflammation and immunological dysregulations, thus proposing a variety of so-called possible neuroimmune biomarkers of mental illnesses and PTSD [9,10,11].

Particularly, the HPA axis has been widely investigated in PTSD: stress exposure causes the release of corticotropin-releasing factor (CRF) and vasopressin from the paraventricular nucleus of the hypothalamus to stimulate the anterior pituitary gland, which in turn secretes the adrenocorticotropic hormone (ACTH) into the systemic circulation. The ACTH induces the release of glucocorticoids, especially cortisol, from the cortical part of adrenal glands; at the same time, stressful situations induce the release of catecholamines (epinephrine and norepinephrine) from the medulla of the adrenal glands. ACTH, cortisol and catecholamines responses depend on the type and intensity of the stressor [5,9,10].

Increasing evidence has been reported on the possible role of inflammation and immunological dysregulations in PTSD pathogenesis, so that pro-inflammatory cytokines are thought to relevantly contribute to the illness presentations, for instance, through the activation of the NF-κB and P38MAPK signal path. Particularly, alterations of interleukin-6 (IL-6), interleukin-1β (IL-1β) and tumor necrosis factor-α (TNF-α) as well as interferon-γ (INF- α) have been implicated in impaired processes of synaptic plasticity and neuroinflammation paths, underlying functional and cognitive anomalies related to PTSD [12]. Moreover, the increased levels of non-specific markers of inflammation such as Erythrocyte Sedimentation Rate (ESR) and C-Reactive Protein (CRP), together with the imbalance between reactive oxygen species (ROS) damages and the activity of antioxidant enzymes, such as catalase (CAT) and superoxide dismutase (SOD), have been highlighted [13]. Similarly, a link between PTSD and an increased risk of physical diseases, such as cardiovascular diseases and autoimmune diseases has been described, suggesting that a dysregulated inflammatory component can be a common subset of the condition [14].

Furthermore, proinflammatory cytokines induced by stress and traumatic event exposure have also been implicated in the upregulation of the indoleamine 2,3-dioxygenase (IDO), which is a crucial enzyme in the kynurenine shunt, a main pathway of tryptophan degradation. The activation of IDO results, at least acutely, in the decrease in tryptophan concentration and the increase in several metabolites, including kynurenic and quinoline acids, that have been involved in the NMDA neurotransmission and possible neurotoxicity [5,15,16].

Although the existing literature indicates possible changes in stress neuroimmune and inflammatory biomarkers in PTSD, the results in human models are still conflicting, while data have only focused mainly on IL-6, CRP and the HPA axis. As a consequence, there is no full consensus on their use as biomarkers in clinical practice. The aim of this systematic review was therefore to summarize evidence suggestive of the following:

1. The presence of a dysregulated inflammatory response in individuals with PTSD versus controls, focusing on specific blood inflammatory biomarkers, as IL-1β, TNF-α, IL-6 and INF-γ, as well as on the potential harmful role on endothelial tissue integrity produced by the decreased clearance of ROS and the reduced antioxidant activity, involving CAT, glutathione peroxidase (GPX) and SOD activities;
2. The role of tryptophan metabolism, or the serotonergic and kynurenine pathways, in the understanding of inflammation in PTSD;
3. The usefulness of these molecular patterns as potential biomarkers of this disorder.

## 2. Materials and Methods

### 2.1. Literature Search

A systematic search was conducted in accordance with the PRISMA guidelines [17] and using the electronic databases PubMed, EMBASE and Web of Science. A combination of controlled vocabulary terms, free-text terms and keywords, without filters, restrictions or limitations, was used to identify all potentially eligible records. The basic search string used was (“IL-6” OR “interleukin-6” OR “IL-1β” OR “interleukin-1β” OR “IFN-γ” OR “interferon-γ” OR “kynurenine” OR “quinolinic acid” OR “ROS” OR “Reactive oxygen species” OR “superoxide dismutase” OR “SOD” OR “catalase” OR “CAT” OR “glutathione peroxidase” OR “GPX” OR “tumor necrosis factor-α” OR “TNF-α” OR “tryptophan” OR “melatonin”) AND (“PTSD” OR “Post-Traumatic Stress Disorder” OR “Posttraumatic stress disorder”). All studies from 1 January 1990, to 31 August 2022 were included in the database search.

### 2.2. Eligibility Criteria

The criteria for inclusion of studies in this review were as follows:

1. Human studies;
2. Studies involving subjects with a clinical diagnosis of PTSD;
3. Studies involving a healthy control (HC) group;
4. Studies that included only subjects aged > 17 years;
5. Articles in English.

Because the aim of the study was to investigate possible in vivo biomarkers of PTSD patients, studies focusing on in vitro investigations (e.g., on cytokine gene expression patterns in cell cultures) and studies using animal models were excluded.

### 2.3. Screening and Selection Process

**Figure 1:**
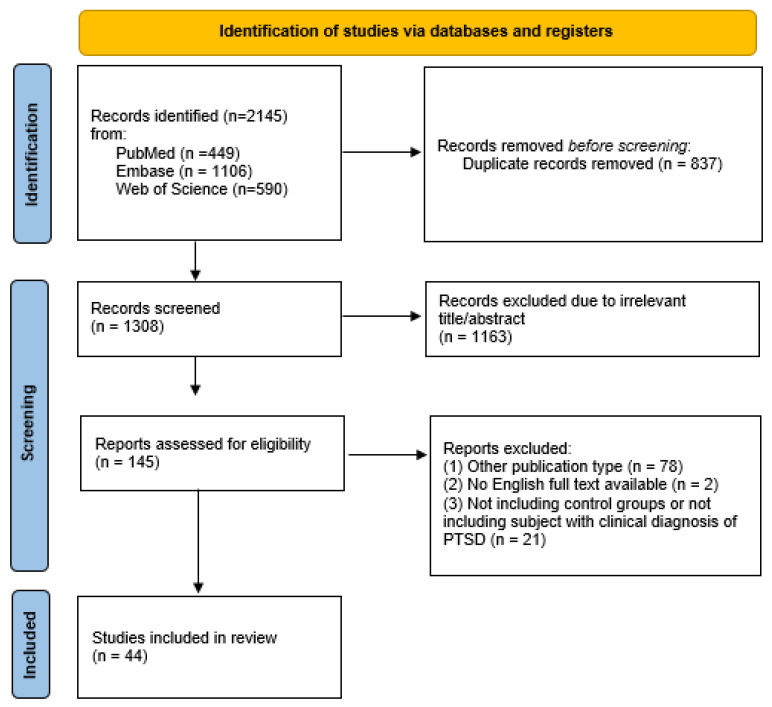
PRISMA flow diagram of the study selection process. PRISMA, Preferred Reporting Items for Systematic reviews and Meta-Analyses; PTSD, Post-Traumatic Stress Disorder.

S.F., first evaluator, and D.G., second evaluator, conducted all phases of the literature selection. The initial database search produced a total of 2145 records. After that, 2000 articles were removed because of their title or abstract, as duplicates (n = 837) or as not relevant (n = 1163); the other 80 records were excluded because of different publication types (n = 78) or because their full text was not available or not in English (n = 2). Subsequently, 21 publications were excluded because they were studies that did not include a control group or a clinical PTSD diagnosis or other eligibility criteria. In addition, all references cited in the selected studies, including reviews and metanalyses, were manually screened. However, no suitable articles emerged from this further research. Finally, 44 articles were included in the present review. The first (S.F.) and second (D.G.) evaluator conducted this selection process independently. Any discrepancy that arose during the categorization phases was discussed and consensus was reached. The overall level of agreement between the two evaluators was good. Any disagreement concerning the inclusion or exclusion of a literature paper in the study was discussed and resolved by a third author (V.D.O.). Inclusion and exclusion decisions are summarized in a flowchart according to PRISMA recommendations [17]. The process of study selection is outlined in this flowchart (Figure 1).

## 3. Results

The search provided the 44 studies included in the review, ranging from 1997 to 2022. Details of each study included in the review are reported in Table 1.

### 3.1. Characteristics of the Study Samples

#### 3.1.1. Population

In the present search, most of the selected works (n = 19, 43.18%) examined samples from the general population, 15 of which included both genders, while four only evaluated females. Veterans were studied in 38.63% of all papers (n = 17), with 10 including only the male gender and seven including both genders. Of note, one of these studies included veterans with rheumatoid arthritis. Finally, 18.8% of the selected papers (n = eight) included other populations, such as earthquake survivors (n = two), war refugees (n = one), women with socioeconomic difficulties (n = one), abused women (n = one), drugs and alcohol abusers (n = one) or psychiatric inpatients (n = two). Regarding the medication status of the patients, 19 studies (43.18%) included a drug-free population. The indication “drug-free” means that patients were recruited if they did not take any drug, including psychotropic medication, or they were under a wash-out period of at least 2 weeks before the beginning of the investigation.

#### 3.1.2. Type of Trauma

In most of the included articles (n = 25; 56.81%), the sample includes individuals who have been exposed to a specific type of traumatic event. Among these, exposure to war seems to be the most common (n = 18; 40.9% of all studies included). Other types of traumas were represented by interpersonal violence (n = two) and earthquakes (n = two); fire/multiple collision car crash (n = one), myocardial infarction (n = one) and urban violence (n = one). However, in 43.18% of the included studies (n = 19), the sample consists of subjects who have experienced various traumatic events.

#### 3.1.3. Mean Ages

The mean age in the PTSD subsample groups was 40.52 years, while the mean age in the health control groups was 39.89 years. The mean ages of the populations were not available in four studies.

### 3.2. PTSD Diagnosis

To assess PTSD, 37 studies (84.09%) only used a scale. The most utilized scale was the Clinician-Administered PTSD Scale (CAPS) (n = 21; 47.72%): particularly, the CAPS was used for DSM-IV (CAPS-IV or CAPS) in 17 studies; the CAPS was used for DSM-5 (CAPS-5) in three studies; and the CAPS was used for DSM-III (CAPS-1) in one study. Eight studies (18.18%) used the DSM criteria to establish the PTSD diagnosis: the DSM-IV was used in four studies; the DSM-IV-R was in one study, the DSM-V was used in two studies and the DSM III-R was used in one case.

Further, seven studies (15.9%) utilized the Structured Clinical Interview for DSM (SCID) criteria: the SCID for DSM-III-R was used in two studies, and the SCID for DSM-IV was used in five studies. The remaining 15 studies (34.09%) assessed PTSD by means of other kinds of psychometric instruments, such as the Mini International Neuropsychiatric Interview (MINI, three studies), the 10th edition of the International Classification of Diseases (ICD-10, four studies), the 9th edition of International Classification of Diseases (ICD-9, one study), the Post-traumatic Stress Diagnostic Scale (PDS, two studies), the Perceived Stress Scale (PSS, one study), the Impact of Event Scale-Revised (IES-R, two studies), the Post-traumatic Checklist Scale (PCLS, one study) and the Composite International Diagnostic Interview (CIDI, one study).

### 3.3. Biomarkers

#### 3.3.1. Biological Sample

In 24 studies (54.54%) the biochemical markers were analyzed in serum, while plasma was investigated in 14 studies (31.81%). In the remaining eight studies (18.18%) other types of biological matrices were collected, particularly whole blood (four studies) liquor (three studies) and saliva (two studies).

#### 3.3.2. IL-6

IL-6 serum or plasma concentrations were investigated in 34 studies. In 20 studies (58.82%), no significant differences were found between IL-6 concentrations in PTSD and HC groups. In 13 studies (38.23%), a significantly higher plasma concentration of IL-6 was reported in PTSD patients compared with control subjects. Only one study (2.94%) detected significantly lower plasma levels of IL-6 in the PTSD group. In addition, one study measured the concentration of IL-6 in CSF; it showed a significantly higher IL-6 amount in the liquor of PTSD patients.

#### 3.3.3. IL-1β

The measurements of IL-1β were obtained from 14 different studies. Seven of them (50%) showed higher serum and plasma concentrations in PTSD patients compared to the HC group, while only one (7.14%) study reported a significantly lower plasma concentration of IL-1β in PTSD than the HC group. Further, no significant difference was found in six studies (42.86%).

#### 3.3.4. TNF-α

In 12 (52.17%) of the 23 studies that analyzed this outcome, higher TNF-α levels were found in the PTSD group compared to HC. There were no significant differences in the remaining 11 studies (47.83%).

#### 3.3.5. IFN-γ

IFN-γ concentrations were investigated in 12 studies. Significantly higher concentrations of IFN-γ in blood and serum samples in PTSD patients than HC were described in three studies (25%). Conversely, no significant differences emerged in nine studies (75%).

#### 3.3.6. Kynurenine and Tryptophan

There was no study investigating kynurenine and tryptophan levels independently. One included study analyzed the serum kynurenine/tryptophan ratio, but no statistically significant differences were highlighted between PTSD patients and HC groups.

#### 3.3.7. Melatonin

Melatonin concentrations were investigated in one study which evidenced lower nocturne-melatonin salivary levels in PTSD patients with respect to HC, despite no statistical differences in the 24 h melatonin concentration were found in the two groups.

#### 3.3.8. Superoxide Dismutase (SOD)

SOD levels were investigated in two studies: one study showed a lower serum SOD concentration in PTSD patients than HC, while no significant plasma SOD activity difference between the two groups was reported in the other study.

#### 3.3.9. Catalase (CAT)

Measurements of CAT were extracted from two studies: one of them showed a significantly higher serum level of CAT in PTSD patients rather than HC, while no difference in the two groups was detected in the other one, where serum CAT levels were measured.

#### 3.3.10. Glutathione Peroxidase (GPX)

The level of GPX was investigated in two studies, both showing a significantly lower serum concentration in PTSD patients with respect to the controls.

#### 3.3.11. ROS/Quinolinic Acid

No studies comparing ROS or Quinolinic Acid levels in PTSD patients with respect to HC were found.

## 4. Discussion

In recent years, increasing interest has been devoted to the possible pathophysiological pathways underlying PTSD. The hypothalamic–pituitary–adrenal (HPA) axis, besides the autonomic nervous system, the monoaminergic transmission system, inflammation and immunological dysregulations, represent key systems hypothesized to be involved in the development of the aforementioned disorder; however, results in human models are still inconsistent [5,9,10,13,14]. As a result, there is no uniform consensus on the use of these mediators as possible biomarkers in clinical practice. Particularly, this review focused on the presence of evidence supporting a dysregulated inflammatory response in individuals with PTSD compared with controls. In this framework, it should be pointed out that, in accordance with the classification proposed by Davis et al., 2015 [62], the studies included in the present review were mostly examining biomarkers of PTSD status, while only a few assessed biomarkers of both trait and disease staging and/or biomarkers of drug response in PTSD (see Table 1).

Specifically, we have targeted some peripheral biomarkers, prevalently those that have been involved in the neuroendocrine response to stress and coping, commonly investigated in other mental illnesses and, therefore, defined as neuroimmune biomarkers [9,10]. These are the main cytokines belonging to the innate immune arsenal such as the aforementioned IL-1β, TNF-α, IL-6 and INF-γ. Furthermore, if considering the interrelationships existing between neuroinflammation, stress and metabolic adaptation to stress after the activation of the sympathetic system and HPA axis, the potential damaging role resulting from impaired ROS clearance and antioxidant activity was also investigated herein, as part of this same response. Thus, studies including the activities of CAT, glutathione peroxidase (GPX) and SOD, the first-line antioxidant enzymes, were comprised in the analysis. Finally, the possible role of inflammatory-altered tryptophan and serotonergic metabolism leading to the kynurenine signaling pathways and the accumulation of potentially neurotoxic metabolites in PTSD, was included.

In regards to IL-6 and INF-γ, most of the included studies [18,20,23,24,25,27,29,34,35,38,40,43,44,45,48,50,52,56,59,60] showed no significant differences between subjects with PTSD and HC. Likewise, differences in IL-β and TNF-α levels between subjects with PTSD and HC were found in slightly more than half of the studies examined. Although the “low-grade inflammation model” [33,35,63] is widely accepted in the scientific community, the results of the present review confirmed conflicting data about the role of pro-inflammatory cytokines in individuals with PTSD. Although some studies did not find significant statistical differences in IL -6 levels between the PTSD group and HC, important elements identified included an upward trend in IL6 [24], the isolated loss of the biphasic plasma peripheral IL-6 circadian pattern with attenuated plasma circadian variability in PTSD compared with HC [23], a positive correlation with symptom severity [34] and higher IL-6 salivary levels as a signal of anticipatory anxiety in the whole sample. This might suggest that the statistical result was influenced by the small number of the sample, resulting in type II error [64]. In addition, the heterogeneity of the studies (e.g., presence or absence of a polytrauma, isolated disorder, acute stress vs. spontaneous assessment) could explain the different results.

Further, scant data are available on the possible role of antioxidant and redox systems in PTSD. Only two studies [28,37] compared the serum levels of SOD in PTSD patients with those of HC, while two studies [37,41] explored the levels of CAT and two studies explored the GPX ones [28,53]. Although results on SOD and CAT were contradictory, both studies on GPX indicated its reduction in serum levels in subjects with PTSD compared to controls. It is noteworthy that, in the presence of oxidative stress, glutathione and other circulating proteins called thiols (P-SH) can be oxidized with a reversible process [65]. Thiol-based redox systems protect cells and organisms against ROS, maintain redox homeostasis and contribute to redox regulation [65,66,67,68], and data suggest that an imbalance in this system could be present in PTSD subjects.

The research yielded no study analyzing tryptophan and kynurenine levels independently, while only one study examined the ratio between kynurenine and tryptophan in serum, with no differences between PTSD patients and HC groups [44]. However, according to currently proposed biological models, proinflammatory cytokines seem to be involved in the upregulation of indoleamine 2,3-dioxygenase (IDO), which is a critical enzyme in the kynurenine shunt [5,15,16]. The activation of IDO leads, at least acutely, to a decrease in the tryptophan concentrations (and indole-conserving pathway metabolites), with an increase in several kynurenine metabolites (including kynurenic and quinolinic acid) which have been associated with NMDA neurotransmission and possible neurotoxicity [5,15,16]. The kynurenine shunt has acquired much interest in stress research and PTSD pathophysiology since it is the main metabolic route of free tryptophan and is a multi-branched pathway regulated by the HPA axis and cytokines [69,70]; it also contributes to balancing tryptophan, serotonin and melatonin amounts in the body, producing a variety of adaptogen derivatives, such as kynurenic acid, anthranilate, picolinic acid, quinolinic acid and the key energy metabolism coenzyme, NAD^+^ [69,70]. Among these compounds, kynurenine and quinolinic acid are particularly noteworthy because they are two main intermediates of the niacin/NAD+ branch, which links the glutamatergic neurotransmission with metabolism and redox reactions [70]. The essential amino acid tryptophan is also the precursor of the hormone melatonin by the indole-conserving pathway, which could be reduced when the kynurenine shunt is induced by inflammation [68]. It is relevant to investigate any changes in melatonin in patients with PTSD, since it regulates the sleep–wake circadian rhythm and sleep disturbances are part of the core symptoms of PTSD [1]. Nevertheless, only one study on this topic emerged [19], reporting the nocturnal melatonin levels in saliva of PTSD patients were lower than those of HC, although no differences in the 24- h melatonin concentration were found. Further studies seem to be necessary to explore this metabolic pathway in depth and its possible role in PTSD.

This review showed the current lack of data on the role of inflammation in subjects with PTSD and on possible biological biomarkers in the human population. Moreover, the most reported traumatic event was the war experience, as the studied sample consisted of veterans or war refugees. It is important to expand the data on different types of traumas to assess whether different events may elicit different pathophysiological responses. Finally, the use of psychotropic drugs could alter peripheral levels of the mediators examined, but less than half of the studies investigated a drug-free population.

In discussing our results, some limitations must be considered. First, we included only English language articles in our selection. Second, some of the included studies have small sample sizes, which may affect the statistical power of the study itself. Finally, the presence of other psychiatric or organic comorbidities as possible exclusion criteria was not always reported in the selected articles, nor were other variables (e.g., premorbid personality, family history, alcohol use), albeit their presence could have influenced the values of the mediators studied.

## 5. Conclusions

Scant and often conflicting data are currently available on the etiopathogenetic mechanisms and possible biomarkers in PTSD subjects. Despite the growing interest in this field, the present research suggests the need for further studies in human samples to deepen the knowledge on the role of inflammation in PTSD pathogenesis, to clearly define potential biomarkers that could be used in clinical practice.

## Figures and Tables

**Table 1:** Characteristics of the included studies.

Study	Years	Country	N Sample		Population	Type of Trauma	Mean Age (PTSD/HC)	Measured Markers	Biologic Sample	Assessment	Main Findings
			Total	PTSD							
Mehta et al. [18]	2020	USA	56	18	Women with socioeconomic difficulties; drug-free	Various	38.8/40.1	IL-6; IL-1β; TNF-α	Plasma	PSS	No difference, but IL-6 was among predictors of MRI striatum-PFC images in most traumatized women
Paul et al. [19]	2019	Canada	14	7	Veterans; controlled psychotropic drug assumption	War	37.57/34.14	Melatonin	Salivary	CAPS-V	↓ Nocturne-melatonin in PTSD patients
Wang et al. [20]	2019	China	187	51	Earthquake Survivors; no anti-inflammatory drugs	Earthquake	48.8/49.96	TNF⍺; IL-6; INF-γ, IL-β	Serum	PCLS	↑ TNF-α and IL-1β in PTSD patients
Imai et al. [21]	2018	Japan	105	40	General population (female); 27.5% were taking psychotropic drugs	Various	38.3/36.4	TNF-α; IL-6; IL-1β	Serum	PDS; IES-R	↑ IL-6 in PTSD patients
Imai et al. [22]	2019	Japan	129	56	General population (female); most were receiving psychotropic drugs	Various	39.2/35.6	TNFα; IL-6	Serum	PDS; IES-R	↑ IL-6 in PTSD patients
Agorastos et al. [23]	2019	USA	35	12	Veterans (male); drug-free	War	27.3/31.7	IL-6	Liquor and plasma	CAPS; SCID-I	No difference, but disrupted circadian IL-6 rhythm
Kim et al. [24]	2020	USA	30	13	Veterans; drug-free	War	40.1/35.0	IL-6	Liquor	PTSD CAPS-IV	No difference, but trend towards ↑ IL6 (*p* = *0*.08)
Kuffer et al. [25]	2019	USA	85	43	General population; drug-free	Various	30.63/30.48	TNF-α; IL-6	Plasma	CAPS	No difference
Brahmajothi et al. [26]	2020	USA	40	20	Veterans; no information about psychotropic drug treatment	War	Not available	IL-6; TNF-α	Plasma	CAPS	↑ TNF-α and IL-6 in PTSD patients
Maloney et al. [27]	2019	USA	1460	170	Veterans with Rheumatoid Arthritis; assumption of anti-arthritis drugs	War	59.3/64.9	IL-1β; IL-6; INF-γ; TNFα	Serum	ICD9	↑IL-1β in PTSD patients
Borovac et al. [28]	2015	Croatia	80	50	Veterans (male); under treatment with sertraline	War	47.1/46.2	eSOD; eGPX	Serum	DSM-IV; ICD10	↓ eSOD and eGPX in PTSD patients
Gola et al. [29]	2013	Germany	60	35	Refugees; 37/31% were taking psychotropic medications	War	32/29	IL-6; TNF-α	Plasma	CAPS	No difference
Vidovic et al. [30]	2011	Croatia	64	39	Veterans (male); drug-free	War	38.5/32.6	IL-6; TNF-α	Serum	CAPS	↑ TNF-α and IL-6 in PTSD patients
Oganesyan et al. [31]	2009	Armenia	62	31	General population; drug-free	Various	42/39	IL-6; IL-1β; TNF-α	Serum	ICD-10	↑ TNF-α, IL-1β and IL-6 in PTSD patients
Von Kanel et al. [32]	2010	Switzerland	44	15	General population; in both groups the 10–13% was taking antidepressants	Myocardial infarction	58.3/58.6	IL-6	Plasma	CAPS	↑ IL-6 in PTSD patients
Hoge et al. [33]	2009	USA	76	28	General population drug-free	Various	41.2/41.7	INF-γ	Plasma	SCID DSM IV	No difference
Song et al. [34]	2007	China	64	34	Earthquake survivors, drug-free	Earthquake	40.4/37.6	IL-6	Serum	DSM IV	No difference;Positive correlation with symptom severity
Von Kanel et al. [35]	2007	Switzerland	28	14	General population; drug-free	Various	33/33	IL-6; IL-1β; TNF-α	Plasma	CAPS	↑ TNF-α and IL-1β in PTSD patients
Woods et al. [36]	2005	USA	94	39	Abused women; no information on psychotropic drug treatment	Interpersonal violence	45.2/46	INF-γ	Blood	DSM-IV-R	↑ IFN-γ in PTSD patients
Tezcan et al. [37]	2003	Turkey	28	14	General population; drug-free	Various	32.48/29.88	SOD; CAT	Plasma	CAPS	No difference
Baker et al. [38]	2001	USA	20	11	Veterans (male); drug-free	War	42.2/41.3	IL-6	Liquor and plasma	SCID DSM-III-R	↑ Liquor IL6 in PTSD patients, no differences in plasmatic IL-6
Maes et al. [39]	1999	Belgium	45	13	General population; no information on psychotropic drug treatment	Fire/a multiplecollision car crash	47/45.3	IL-6	Serum	DSM III-R	↑ IL-6 in PTSD patients
O’Donovan et al. [40]	2014	USA	205	40	Veterans; no information on psychotropic drug treatment	War	42.12/45	IL-6	Plasma	CAPS	No difference;
Oglodek et al. [41]	2016	Poland	460*	60	General population; drug-free	Various	46.8/42.4	TNF-α, GPX-1	Serum	ICD10	↑ TNF-α, ↓ GPX-1 in PTSD and in PTSD + depressive patients
Blessing et al. [42]	2017	USA	166	83	Veterans (male); controlled psychotropic drug assumption	War zones exposition	33/32.5	IL-6, TNF-α	Serum	CAPS	↑ TNF-α and IL-6 in PTSD patients
Jergovic et al. [43]	2015	Croatia	101	69	Veterans (male); under psychotropic medication and treatment-resistant	War	47.12/45.56	IL-1β; IL-6; TNF-α; INF-γ	Serum	ICD10	No difference
Neupane et al. [44]	2017	Norway	187	32	Drugs and alcohol abusers; no information on psychotropic drug treatment	Various	33.1/35.9	IL-6; TNF-α; INF-γ Kynurenine/tryptophan	Serum	CIDI and DSM-IV	No difference
Bruenig et al. [45]	2018	Australia	299	159	Patients of the Greenslopes Hospital of Australia; controlled psychotropic drug assumption	Various	68.47/69.23	IL-1β; IL-6; TNF-α; INF-γ	Serum	CAPS-5	No difference
Bersani et al. [46]	2015	USA	121	56	Veterans (male); controlled psychotropic drug assumption	War	33.91/32.81	IL-1β; IL-6; TNF-α; INF-γ	Serum	CAPS 5	↑ TNF-α and IL-6 in PTSD patients
Lindqvist et al. [47]	2017	USA	61	31	Veterans (male); controlled psychotropic drug assumption	War	31.2/30.8	IL-6; TNF-α; INF-γ	Serum	CAPS	↑ IL-6 in PTSD subjects
Lindqvist et al. [48]	2014	USA	104	52	Veterans (male); controlled psychotropic drug assumption	War or combat-exposed	34.1/33.7	IL-1β; IL-6; TNF-α; INF-γ	Serum	CAPS	↑ TNF-α, IFN-γ in PTSD patients
De Oliveira et al. [49]	2018	Brazil	82	41	General Population; drug-free	Various	27.32/27.2	IL-6	Serum	MINI according with DMS-IV	↑ IL-6 in PTSD patients
Teche et al. [50]	2017	Brazil	60	30	General population; no information on psychotropic drug assumption	Urban violence	Not available	IL-6	Serum	MINI	No difference
Oglodek et al. [51]	2015	Poland	220	120	General population; drug-free	Various	Not available	Il-6	Plasma	DSM 5	↑ IL-6 in PTSD patients
Jergovic et al. [52]	2014	Croatia	47	30	Veterans; Psychotropic drug assumption	War	45.9/47.2	INF-γ, TNF-α, IL-6	Serum	CAPS	↑ IFN-γ in PTSD patients
Oglodek et al. [53]	2017	Poland	460	60	General population; drug-free	Various	45.2/42.4	CAT	Serum	DSM5	↑ CAT in PTSD patients
Tucker et al. [54]	2004	USA	107	86	General population; drug-free	Various	Not available	IL-1β	Serum	SCID-IV and CAPS-I	↑IL-1β in PTSD patients
Spivak et al. [55]	1997	Israel	38	19	Veterans (male); drug-free	War	25.3/31	IL-1β	Serum	SCID-P (DSM-III-R)	↑IL-1β in PTSD patients
Park et al. [56]	2017	USA	28	14	Veterans; controlled psychotropic drug assumption	War	34.4/32.2	IL-6	Blood	CAPS-IV	No difference
Guo et al. [57]	2012	China	100	50	General population; subgroups under psychotropic medication	Various	42/41	IL-6-TNF-α	Serum	DSM-IV	↑ TNF-α and IL-6 in PTSD patients
Dalgard et al. [58]	2017	USA	27	16	General population; drug-free	Various	31.5/29.5	IL-1β; IL-6; TNF-α; INF-γ	Plasma	SCID-I; CAPS-IV	↑ TNF-α, ↓ IL-1β in PTSD patients
Newton et al. [59]	2014	USA	63	15	General population (female); no exclusion of psychotropic drug assumption	Interpersonal violence	53.55/54.9	IL-6	Saliva; Plasma	CAPS-IV	No difference; but ↑ IL-6 salivary levels as a signal of anticipatory anxiety in the whole sample
Renner et al. [60]	2022	Germany	53	17	General population(female); drug-free	Various	46.88/41.94	IL-6	blood	SCID-IV	No difference
Toft et al. [61]	2021	Norway	81	33	Patients of Modum Bad Psychiatric Center; no exclusion of psychotropic drug assumption	various	39.6/41.9	IL-1β; TNF-α	blood	MINI	↑ IL-1β and TNF-α in PTSD patients

## Data Availability

All data generated or analyzed during this study are included in this published article.
